# Species identity and behavior of cave‐dwelling tree hyraxes of the Kenyan coast

**DOI:** 10.1002/ece3.9693

**Published:** 2023-01-15

**Authors:** Hanna Rosti, Henry Pihlström, Norbert Rottcher, Simon Bearder, Lucas Mwangala, Marianne Maghenda, Jouko Rikkinen

**Affiliations:** ^1^ Finnish Museum of Natural History University of Helsinki Helsinki Finland; ^2^ Organismal and Evolutionary Biology Research Programme, Faculty of Biological and Environmental Sciences University of Helsinki Helsinki Finland; ^3^ Molecular and Integrative Biosciences Research Programme, Faculty of Biological and Environmental Sciences University of Helsinki Helsinki Finland; ^4^ Department of Botany Pwani University Kilifi Kenya; ^5^ Nocturnal Primate Research Group Oxford Brookes University Oxford UK; ^6^ Programme and Planning Academic Research and Outreach Division TAITAGIS Taita Taveta University (TTU) Voi Kenya; ^7^ Department of Agricultural Sciences School of Agriculture Earth and Environment Sciences TAITAGIS Taita Taveta University (TTU) Voi Kenya

**Keywords:** bioacoustics, nocturnal mammals, PAM passive acoustic monitoring, sacred forests, thermal imaging

## Abstract

We surveyed tree hyrax populations living in forests, limestone rocky formations, and caves in coastal Kenya to identify the species and estimate the threat‐level populations are in. Tree hyrax vocalizations were recorded in three different habitats with passive acoustic monitoring (PAM) for a total of 84 h in January and February 2022. We also observed tree hyrax behavior with thermal imaging camera and photographed individuals. Tree hyraxes in coastal Kenya are vocally active throughout the night, with most calls emitted between 23.00 and 04.00. We identified four different calls: snort, hac, hac ping‐pong, and wheeze. Their calling range is between 220 and 15,000 Hz. Calls of tree hyraxes from the coast of Kenya were compared with calls stored by the Oxford Brookes University's Nocturnal Primate Research Group and identified as eastern tree hyrax, previously recorded from Tanzania. Here, we present what are, to our knowledge, the first photographs of live *D. validus* from Kenya. These tree hyraxes live in social groups. Due to strong pressure from humans, conservation measures are necessary to prevent the extinction of these isolated *D. validus* populations in Kenya.

## Graphical abstract

In this article, we describe the appearance, behavior, acoustic communication, and habitats of Eastern tree hyraxes (*Dendrohyrax validus*) from the coast of Kenya. Outside Shimba Hills National Reserve, tree hyraxes are extremely endangered and survive in sacred caves and rocky outcrops.

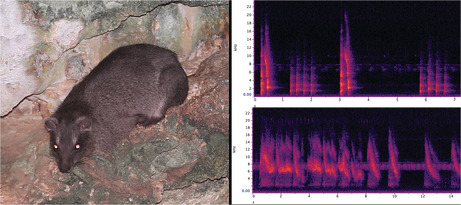

## 1 INTRODUCTION

Tree hyraxes are distant relatives of elephants and sirenians (Nishihara et al., 2005; Poulakakis & Stamatakis, 2010; Seiffert, 2007; Springer et al., 1997; Stanhope et al., 1998; Tabuce et al., 2008; van Dijk et al., 2001). Tree hyraxes are browsers and use the foliage of trees as their main food source (Gaylard & Kerley, 1997; Kundaeli, 1976; Milner & Harris, 1999a; Roberts et al., 2013). They also need hollow trees or other hideouts, where they spend most of the day (Gaylard & Kerley, 2001; Kundaeli, 1976; Milner & Harris, 1999b; Opperman et al., 2018). Tree hyraxes use woody climbers for moving between trees, and dense growths of tangled lianas provide hideouts for the animals (Allen & Loveridge, 1927; Kingdon, 1971; Rosti et al., 2022). Tree hyraxes are vocally very active, and their acoustic communication can be used to distinguish between different species (Hoeck et al., 2014; Oates et al., 2022; Roberts, 1999, 2001; Rosti et al., 2020). Previously, tree hyraxes have been thought to be solitary (Kundaeli, 1976), but recent findings from the Taita Hills in Kenya suggest that they can be highly social (Rosti et al., 2022). Tree hyraxes have short legs, and their ability to migrate between isolated forest fragments is poor. Lawes et al. (2000) studied tree hyrax patch occupancy and found that the probability of occupancy of a forest patch by tree hyraxes was zero if the distance between forest patches was more than 1.5 km.

The number of extant tree hyrax species is controversial. Traditionally, three species have been recognized: western tree hyrax (*D. dorsalis*), southern tree hyrax (*D. arboreus*), and eastern tree hyrax (*D. validus*) (Hahn, 1934; Kingdon, 1971; Meschke, 2019). Recently, a fourth species, the Benin tree hyrax *D. interfluvialis*, was described from West Africa (Bearder et al., 2015; Oates et al., 2022). However, the exact ranges of the different tree hyrax species are poorly known and many questions about their taxonomy are far from settled, suggesting that undescribed species may still await scientific discovery (Hoeck, 2017; Oates et al., 2022; Roberts et al., 2013).

**FIGURE 1:**
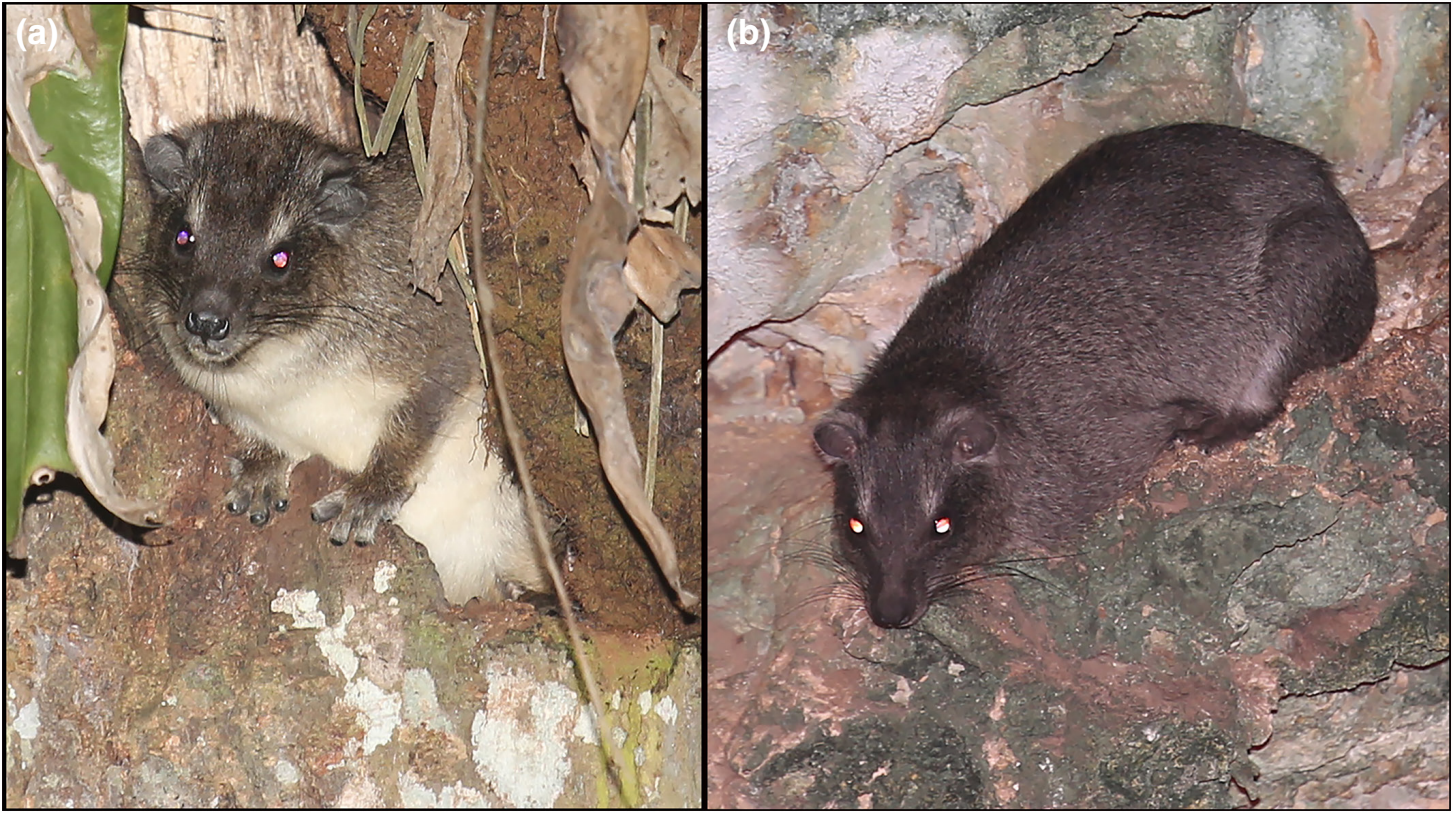
(a) Eastern tree hyrax (*Dendrohyrax validus*) from Shimba Hills, Kenya. (b) *D. validus* from limestone cave in Vipingo near Mombasa, Kenya (photos by Hanna Rosti and Norbert Rottcher 2022).

The first account of tree hyraxes living in caves at the Kenya coast was published by Seibt et al. (1977), but this report has been largely overlooked. The discovery was fortuitous, as the authors were actually looking for fruit bats that roost in the caves. They observed tree hyraxes, recorded their vocalizations, and even collected some “mummified” remains and a complete skull of an adult male hyrax. Based on this material, Seibt et al. (1977) identified the cave‐dwelling hyraxes as eastern tree hyrax *Dendrohyrax validus* but did not assign it to any subspecies. Their record was the first report of this species from Kenya; previously, eastern tree hyrax was only known from Tanzania. The eastern tree hyrax *Dendrohyrax validus* (Figure 1a,b) has a short, smooth, dark brown coat. According to the IUCN's assessment (Hoeck et al., 2014), *D. validus* has a fragmented distribution in East Africa (Kingdon, 1971). As is the case of tree hyraxes generally, the subspecies‐level taxonomy of the eastern tree hyrax taxonomy is controversial (see, e.g., Meschke, 2019, Rosti et al., 2020).

Tree hyrax population density is usually estimated by analyzing the calling rate, as their sightings are rare (Rosti et al., 2022). The highest population densities of tree hyraxes have been found in large and intact natural forests (Topp‐Jørgensen et al., 2008). Smaller forest size is usually linked with high levels of human disturbance such as the removal of trees and branches for timber and firewood, as well as poaching. When forest size decreases, calling patterns, and calls/hour change significantly, probably because tree hyraxes try to avoid being noticed by humans (Rosti et al., 2022).

*Dendrohyrax validus* population decline has been significant over the course of the last two decades due to hunting and habitat loss (Hoeck et al., 2014). In the Tanzanian Eastern Arc Mountains, tree hyraxes are threatened by hunting and snaring (Allen & Loveridge, 1927; Cordeiro et al., 2005). Even though the species is still locally abundant in some protected sites, sufficient data for accurately estimating population numbers are not available; therefore, *D. validus* is classified as Near Threatened by the IUCN (Hoeck et al., 2014).

The purpose of this study is taxonomical confirmation and disturbance‐level estimation of previously unstudied populations of *Dendrohyrax validus* living in limestone caves and rocky outcrops on the coast of Kenya. We present photographs and videos (Figure S1) of *D. validus*, describe their acoustic communication, calling patterns, and social behavior, and also estimate current conservation needs.

## 2 METHODS

### 2.1 Study sites

**FIGURE 2:**
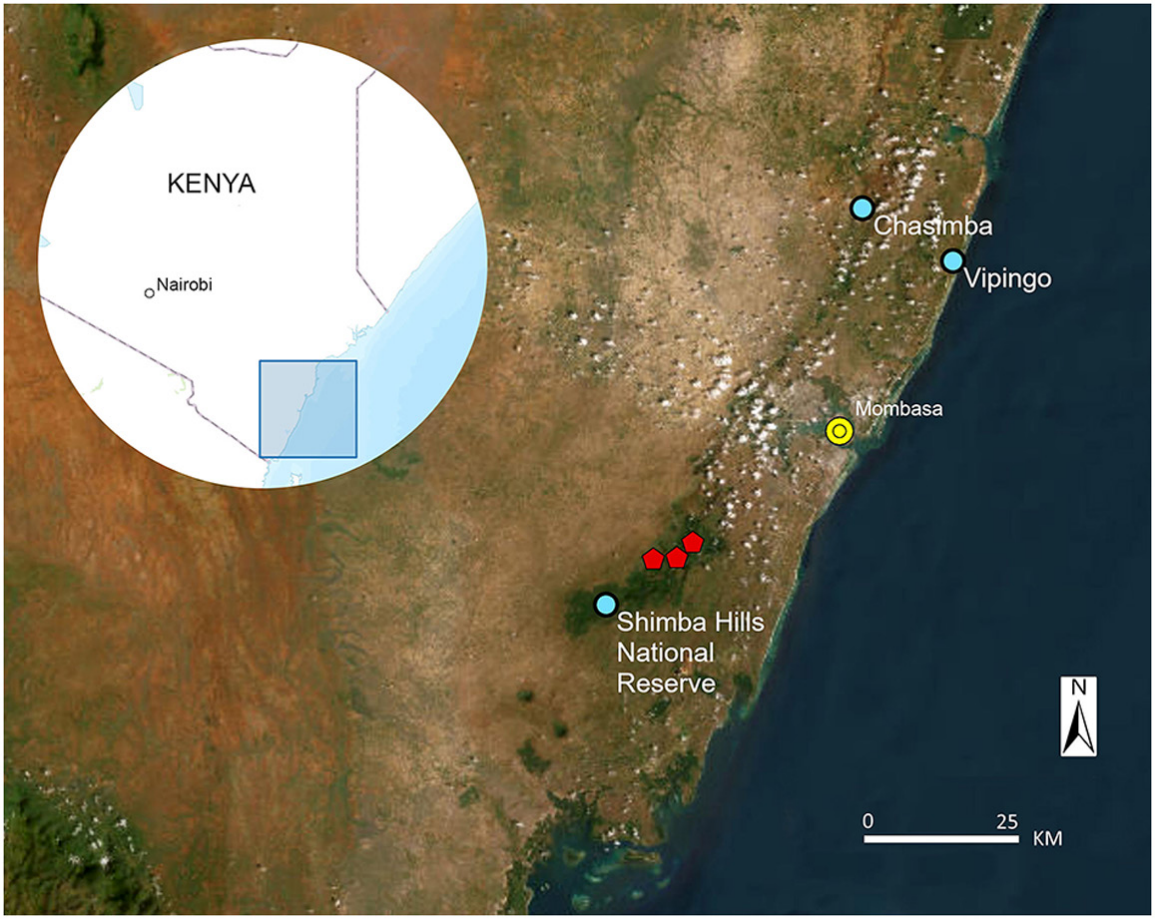
Study locations at the coast of Kenya. Shimba Hills had three study sites.

**FIGURE 3:**
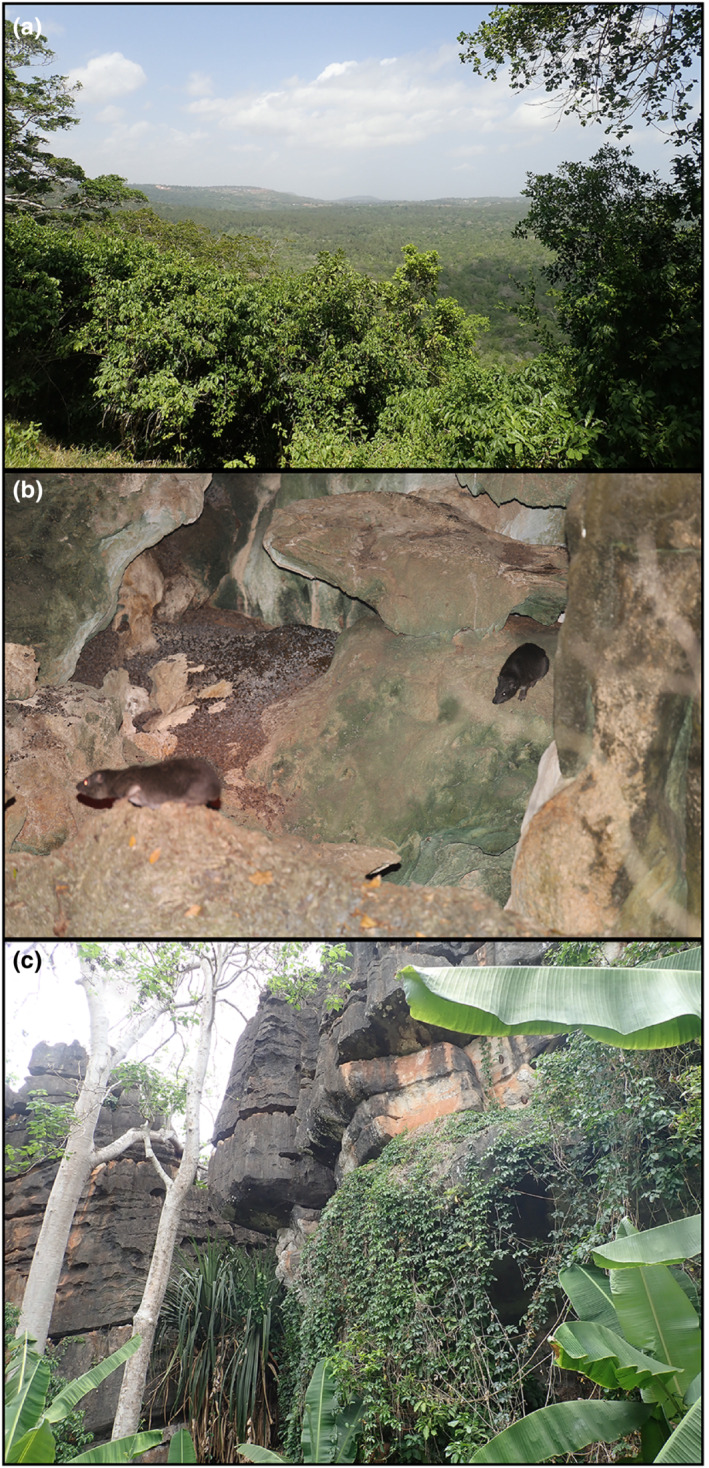
(a) Natural moist forest in Shimba Hills National Reserve. (b) Limestone cave at Vipingo. (c) Rocky formation in Chasimba (photos by Hanna Rosti and Norbert Rottcher 2022).

We were able to locate and visit three tree hyrax habitats on the Kenya coast between Jan 26 and Feb 5, 2022 (Figure 2). The first study site, the Shimba Hills National Reserve, 4.214 S, 39.451 E, has a total area of 192.5 km^2^. The reserve supports forests (Figure 3a) and grassland areas that are kept open by periodic burning. The local tree hyraxes live in trees. The second site, Vipingo (coordinates not published), is on private land. It represents a 25 ha patch of old natural forest with large baobab trees (Figure 3b), surrounded by agricultural lands and villages. The site also has three large sacred limestone caves with bat colonies. The local tree hyraxes live in caves and trees. The third site, Chasimba, 3.739 S, 39.693 E, represents a small (2 ha) rocky outcrop with sacred caves and small trees (Figure 3c), surrounded by agricultural fields and banana groves. The local tree hyraxes live in rocky formations higher up. Unfortunately, the site has lost most of its tree cover and is in imminent threat of being turned into a limestone quarry, which would undoubtedly eradicate the local tree hyrax population, as there are no other suitable habitats nearby. Distance between Shimba Hills and Chasimba is 60 km.

### 2.2 Materials and methods

Tree hyrax species can be identified by their calls (Oates et al., 2022; Roberts et al., 2013). Calling activity and pattern of tree hyraxes correlate with population size and level of human disturbance (Rosti et al., 2022). For recordings, we used AudioMoth automatic recorders (v1.1.0 Open Acoustics Devices) and Song Meter4 (Wildlife Acoustics, Maynard USA). Recordings were made between 19.00 and 07.00. As tree hyraxes are shy, we used a thermal imaging camera, Pulsar Helion 2 XP50 (Yukon Advanced Optics Worldwide, Vilnius, Lithuania) to observe tree hyrax natural behavior during the night. Photographs were taken with Canon EOS 5D Mark III (Canon Inc). For supporting light while taking photographs, we used Fenix TK25 RED (Fenix Lighting) flashlights.

Spectrograms and call analyses were done with Raven Pro 1.6 (Cornell University). Different call types were identified, and clear recordings with no background noise were isolated. Tree hyrax calling activity for each hour was calculated by using the band‐limited energy detection function in Raven Pro 1.6. A band‐limited interactive detector was used with the following settings: min frequency 1000 Hz, max frequency 4500 Hz, min duration 0.01 s, max duration 2 s, min separation 0.23 s. Signal‐to‐noise ratio (SNR) was 70%, and threshold of SNR was 10. False positive calls, (mostly short‐eared greater galago *Otolemur garnettii* calls), were removed from the data set by visually inspecting all spectrograms. We compared our audio recordings with a reference material of tree hyrax vocalizations collected and deposited by the Nocturnal Primate Research Group at Oxford Brookes University, United Kingdom.

We compared our photographs and video material of living tree hyraxes with tree hyrax skins preserved in the collections of the National Museums of Kenya in Nairobi, Kenya, and in the Swedish Museum of Natural History in Stockholm, Sweden. This allowed us to compare the physical appearance of the Kenya coast tree hyraxes with representatives of different populations of other tree hyrax species.

## 3 RESULTS

### 3.1 Appearance

*Dendrohyrax validus* has a dark brown, short, and smooth coat (Figure 1a,b; Figure S1). It has tactile hair on the face and other parts of the body. The eyes of *D. validus* reflect back when photographed with flashlight, indicating that they have a tapetum lucidum membrane behind the retina. To our knowledge, these are the first photos published of *D. validus* taken from live animals in their natural habitats in Kenya.

Collections in Natural History Museum in Stockholm have several *D. validus* specimens from Mt Kilimanjaro, Tanzania similar in appearance (accession numbers 580764, 581158, 582155, 582274, 582417–582419, 582421, 583216, 583268, 583279, and 592420).

### 3.2 Habitats and behavior

In Shimba Hills two of the three AudioMoths placed in mature natural forests did not record any tree hyrax calls, indicating that the animals are not common throughout the region. Two species of galagids (*Otolemur garnettii* and *Paragalago cocos*) were common at all three study sites. In Shimba Hills, we could hear tree hyraxes vocalizing while moving around in the forest, indicating that calling plays a role in group coordination.

In Vipingo, the tree hyraxes clearly live in groups, as we could repeatedly observe as many as nine individuals together. At this site, tree hyraxes were often seen walking on the ground. They used the caves as resting sites, and occasionally the cave‐dwelling animals responded to the calls of others that were vocalizing in nearby trees. In Vipingo, *Otolemur garnettii* was calling actively, but no calls of *Paragalago cocos* were recorded.

In Chasimba, tree hyraxes inhabit a rocky outcrop with vertical cliffs of up to 15 meters. They climb on the rocks and find safe daytime resting sites from the cliffs. At this site, tree hyraxes were the only nocturnal animals caught in our recordings (below 22 kHz), katydids and galagos were absent. Here, as at both other study sites, tree hyraxes defecate in middens.

### 3.3 Calling activity

**FIGURE 4:**
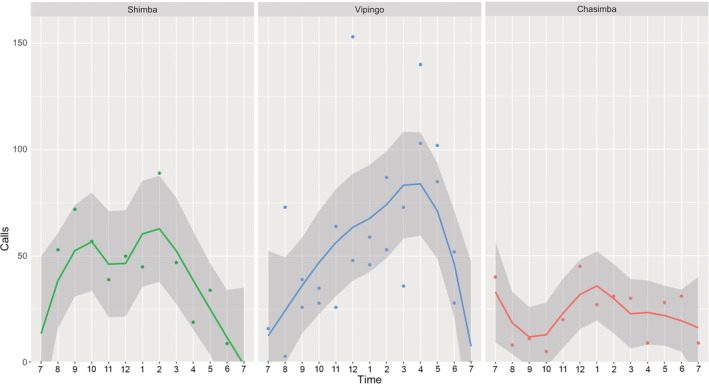
Tree hyrax (*Dendrohyrax validus*) calls per hour from 19.00 until 07.00 in the morning at the study sites in Shimba Hills, Vipingo, and Chasimba.

The data on calling activities were obtained during single recording nights in Shimba Hills and Chasimba, respectively, and two recording nights in Vipingo. At all three sites, tree hyraxes were called most actively between 23.00 and 04.00 (Figure 4). Occasionally, but very infrequently, the animals were also heard calling during the day. In Shimba Hills, the highest calling activity was between 01.00 and 2.00, with 89 calls. Average calling rate in Shimba Hills was 42 calls per hour. In Vipingo, the highest calling activity was between 23.00 and 24.00, with 153 calls. In Vipingo, the average calling rate per hour was 53. In Chasimba, the highest calling activity was between 23.00 and 24.00, with 45 calls. In Chasimba, the average calling rate per hour was 23.

### 3.4 Description and analysis of calls

**FIGURE 5:**
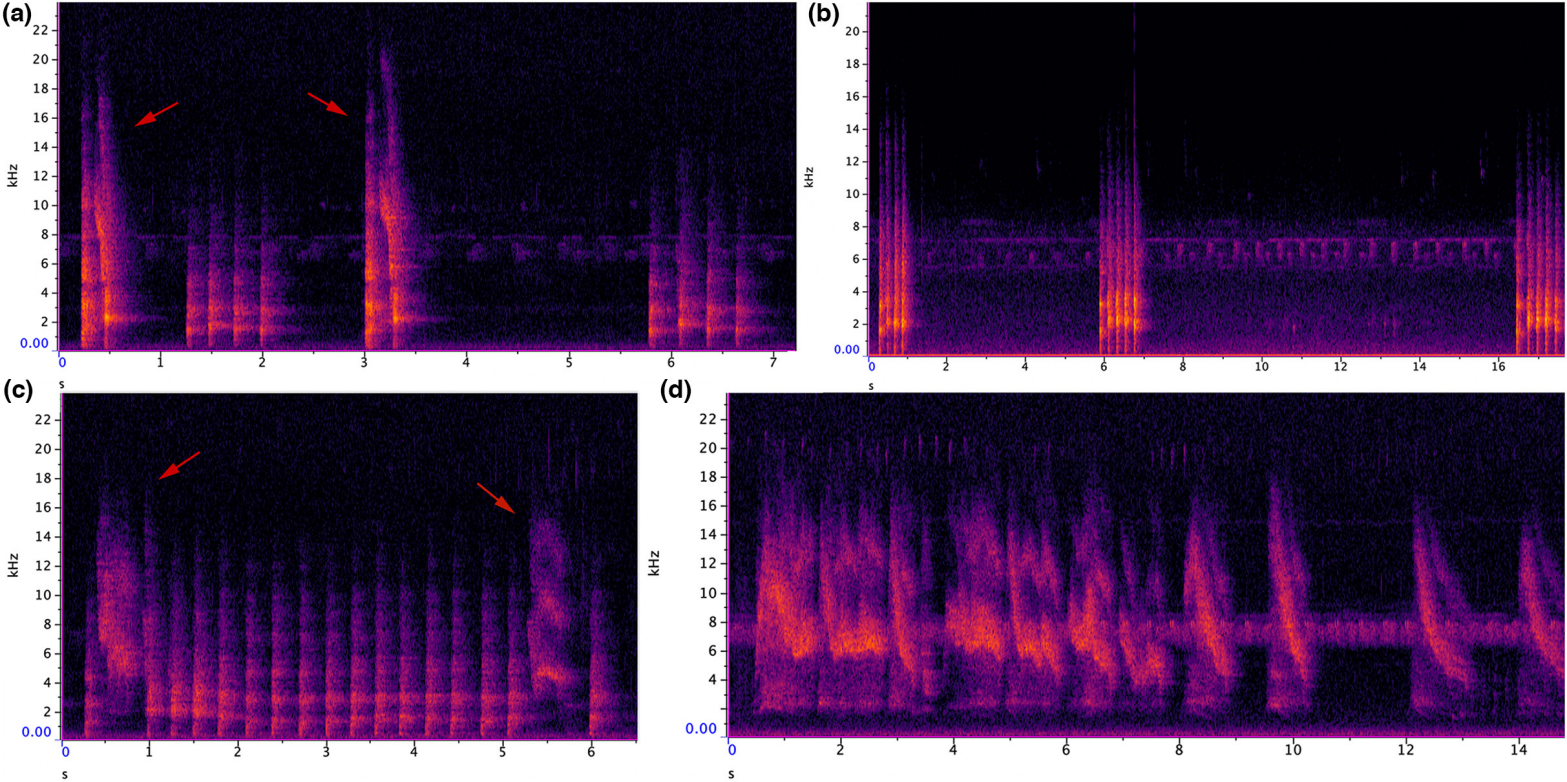
(a) Snort calls (arrows) and hac calls of the tree hyrax (*Dendrohyrax validus*) from Vipingo. (b) Hac calls from Shimba Hills. (c) Hac ping‐pong sequence with two wheezes (arrows) from Vipingo. (d) Wheeze sequence from Vipingo in the Kenyan coast.

*Dendrohyrax validus* calling range is 220–15,000 Hz (Figure 5). Typical calls are short (duration 0.25–1.4 s), although some calls are longer. We could recognize four different calls, even though elements of all of them could often be modified and combined, adding variation to the acoustic communication.

- **Snort call** has average duration of 0.4 s and maximum frequency of 3000 Hz (*n* = 61) (Figure 5a; Audio S1). Second pulse‐like element has a curve that sounds like a chuck with a twist. This call was used at all sites, the example call was recorded from Vipingo.
- **Hac call** is the most commonly used call at all locations (Figure 5b; Audio S2). Average duration of the call is 1.1 s, and maximum frequency is 2100 Hz (n 103). This element is repeated from one to seven times, with the most common number of repeats being four. Hac calls are almost invariably responded to with a hac call. The example call was recorded from Shimba Hills.
- **Hac ping‐pong sequence**. In this call, hac is repeated 10–40 times, and sometimes, such as the example, combined with a wheeze element (Figure 5c; Audio S3). Duration is several seconds (up to 10 s), and maximum frequency is 3100 Hz (*n* = 13). This type of call was only recorded in Vipingo.
- **Wheeze**. This call sounds like a whistle (Figure 5d, Audio S4). Here the element is repeated 17 times. Maximum frequency is 6100 Hz. The call has a variable number of harmonics from 3000 to 12,000 Hz. The call was usually combined with other elements and was recorded alone only once in Vipingo.

## 4 DISCUSSION

*Dendrohyrax validus* was first described by True (1890) from Mt Kilimanjaro in Tanzania. The dark and smooth coat of the tree hyraxes at the Kenya coast corresponds closely to skins of this species in the collections of the Natural History Museum in Stockholm. The museum specimens had been collected from Mt Kilimanjaro, which is located approximately 250 km NW of Shimba Hills. Molecular data are needed to gain a better understanding of relationships between different *D. validus* populations in East Africa.

Southern tree hyraxes, *D. arboreus* are known to inhabit rocky terrain in the Ruwenzori Mountains, Uganda, and in this type of habitat, the animals have been reported to live in colonies (Kingdon, 1971). In some parts of Usambara and Uluguru Mountains, Tanzania, tree hyraxes have been observed to retreat to boulders and rocky outcrops as local forests were cleared (Allen & Loveridge, 1927). Clearly, rock formations and caves can provide suitable resting places for tree hyraxes, and if at least some trees suitable for foraging remain in the area, they may have high value as refugia for dwindling tree hyrax populations.

While tree hyraxes of the Kenya coast are clearly not solitary animals, the social structure of their groups remains unknown. Recent research in the Taita Hills, Kenya, also suggests that tree hyraxes are more social than previously thought (Rosti et al., 2020, 2022). Tree hyraxes call most actively between 24.00 and 04.00 hr. This timing may reflect natural behavior, but it may also be a response to human disturbance (Gaynor et al., 2018). At the Kenyan coast, tree hyraxes sometimes, although rarely, are heard calling during the day. *Dendrohyrax validus* uses several different call types and different calls are commonly graded and combined. Call descriptions may have value for elucidating taxonomic relationships, as different tree hyrax species exhibit differences in vocal communication (Oates et al., 2022; Roberts, 2001; Roberts et al., 2013).

According to Seibt et al. (1977), local people of the Kenya coast, the Giriamas, consider some caves sacred and avoid disturbing bats and other animals that live in the caves (cf. Metcalfe et al., 2010). Seibt et al. (1977) hence suggested that the hyraxes may have received a level of protection from the sacred caves for quite some time, and might thus represent a relic population. We can confirm that the caves are still used by local people for cultural purposes and a population of tree hyraxes still exists around the caves. As the local coastal forests have been almost completely cleared, caves and sacred forest groves have become rare habitat islands, surrounded by open farmland or secondary shrub. Thus, such sites should be prioritized in conservation, not only for cultural reasons but also for their high value in conserving biodiversity (Brandt et al., 2013; Byers et al., 2001; Himberg, 2011; Ormsby & Bhagwat, 2010).

We recommend that the International Union for Conservation of Nature (IUCN) re‐evaluates the distribution and status of *Dendrohyrax validus* by also including these endangered populations on the coast of Kenya. We also recommend that the conservation of these fragmented and isolated populations of tree hyraxes should have high priority in Kenya. Currently, there are plans to start limestone mining in Chasimba, which would likely eradicate the *D. validus* population from the site. Also, more extensive surveys of all possible fragments should be conducted to locate all remaining populations in coastal Kenya for conservation purposes.

## 5 CONCLUSIONS

In coastal Kenya small and isolated populations of *Dendrohyrax validus* survive in patches of remaining coastal forest, and in limestone outcrops and caves. Videos from the caves demonstrate that the eastern tree hyrax is a social species. This vocally active animal has at least four distinct call types and its vocalizations have potential value for taxonomical and behavioral ecology studies. Calling activity is highest in the middle of the night between 23.00 and 04.00. Due to deforestation and mining, these *D. validus* populations are seriously threatened outside Shimba Hills, and urgent conservation efforts should be implemented to ensure the survival of the isolated populations. Effective conservation of the tree hyrax requires the protection of all remaining fragments of natural forest in the region. Mining at these sites should be prevented. Reforestation efforts around small forest patches are also needed and increasing awareness among local people through ecotourism activities is important for the survival of the remaining tree hyraxes.

## AUTHOR CONTRIBUTIONS

**Hanna Rosti:** Conceptualization (equal); data curation (equal); formal analysis (equal); funding acquisition (equal); investigation (equal); methodology (equal); project administration (equal); software (equal); visualization (equal); writing – original draft (equal). **Henry Pihlstrom:** Conceptualization (equal); investigation (equal); validation (equal); writing – original draft (equal); writing – review and editing (equal). **Norbert Rottcher:** Conceptualization (equal); funding acquisition (equal); investigation (equal); visualization (equal); writing – review and editing (equal). **Simon Bearder:** Conceptualization (equal); formal analysis (equal); methodology (equal); supervision (equal); writing – original draft (equal); writing – review and editing (equal). **Lucas Mwangala:** Project administration (equal); writing – review and editing (equal). **Marianne Maghenda:** Project administration (equal); writing – review and editing (equal). **Jouko Rikkinen:** Project administration (equal); resources (equal); supervision (equal); writing – review and editing (equal).

## FUNDING INFORMATION

The author HR was supported by the Kone Foundation Grant 202007604, Tuovinen Foundation, Ripaco, and by the University of Helsinki. The author HP was supported by grants from the Oskar Öflund Foundation and the Waldemar von Frenckell Foundation.

## CONFLICT OF INTEREST

The authors declare no competing interests.

### OPEN RESEARCH BADGES

This article has earned an Open Data badge for making publicly available the digitally‐shareable data necessary to reproduce the reported results. The data is available at https://doi.org/10.5061/dryad.sf7m0cg99.

## Supporting information


Figure S1.

Audio S1.

Audio S2.

Audio S3.

Audio S4.

## Data Availability

The data that support the findings of this study are openly available in Dryad at https://doi.org/10.5061/dryad.sf7m0cg99.
