# Supplementary figures and images for: Species identity and behavior of cave‐dwelling tree hyraxes of the Kenyan coast
Source: Ecol Evol. 2023 Jan 15;13(1):e9693. doi: 10.1002/ece3.9693 (PMC9841124; doi:10.1002/ece3.9693)

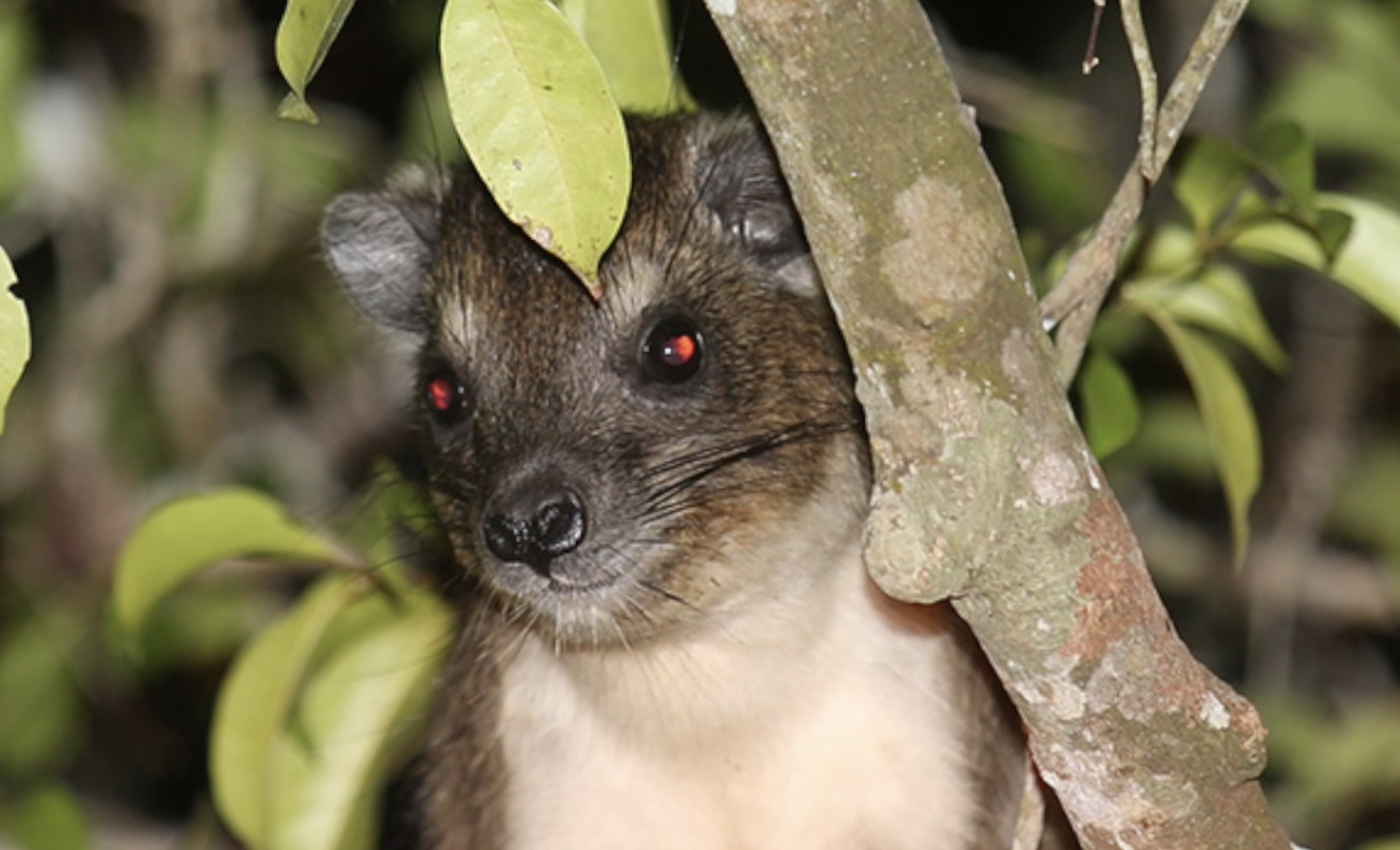

Supplement: Supplementary file 1 — Figure S1. [file ECE3-13-e9693-s002.png]
